# Ivermectin induced Steven–Johnsons syndrome: case report

**DOI:** 10.1186/s13104-017-2500-5

**Published:** 2017-05-08

**Authors:** Desmond Aroke, Diego Nitcheu Tchouakam, Alexis Tazinya Awungia, Sylvester Yari Mapoh, Stewart Ndutard Ngassa, Benjamin Momo Kadia

**Affiliations:** 1 Nkwen Baptist Health Center, Bamenda, Cameroon; 2 Health and Human Development (2HD) Research Group, Douala, Cameroon; 3 Roua District Hospital, Roua, Cameroon; 4 Mboppi Baptist Hospital, Douala, Cameroon; 5 Universite Libre Brussels, Brussels, Belgium; 6 Bali Kumbat Sub-Divisional Hospital, Bali Kumbat, Cameroon; 7 Presbyterian General Hospital Acha-Tugi, Acha, Cameroon

**Keywords:** Ivermectin, Stevens–Johnson syndrome (SJS), Human immunodeficiency virus (HIV)

## Abstract

**Background:**

Stevens–Johnson syndrome is one of the manifestations of mucocutaneous adverse drug reactions. Although antimicrobials are responsible for greater than 50% of these adverse drug reactions, there is no documented case implicating ivermectin as the culprit.

**Case summary:**

A 38 year old adult Cameroonian male presented to our health facility with facial rash, painful oral sores, black eschars on lips and red tearing eyes 3 days following ingestion of ivermectin received during a nationwide anti-filarial campaign. He had no known chronic illness, no known allergies and was not on any medications prior to the campaign. Physical examination revealed discharging erythematous eyes, crusted and blister-like lesions with cracks on his lips and oral mucosa. His laboratory tests were unremarkable but for a positive Human Immunodeficiency Virus (HIV) test. A diagnosis of Ivermectin induced Stevens–Johnson syndrome in a newly diagnosed HIV patient was made. The patient was managed with supportive therapy and the evolution thereafter was favourable.

**Conclusion:**

Stevens–Johnson syndrome is a potential side effect of ivermectin and susceptibility to this adverse effect may be increased in HIV infection.

## Background

Stevens-Johnson syndrome (SJS), a form of mucocutaneous adverse reaction affecting <10% of body surface area is almost always drug related [1–3]. Antimicrobials are thought to be the most common cause of SJS. The antimicrobials often implicated are sulphonamides, penicillins, anti-tuberculoses drugs and anti-retrovirals [4, 5]. Cases of SJS have been reported with other antimicrobials such as fluconazole and streptomycin, not known to be typical causes of SJS [6, 7]. Furthermore, ingestion of paracetamol is implicated in very few cases of SJS [8]. However, to the best of our knowledge, no case of ivermectin induced SJS has been previously described in literature. Studies have also shown that there is an increase occurrence of cutaneous drug reactions seen in patients with human immunodeficiency virus (HIV) infection [9].

We herein report the case of a patient who presented with SJS following ingestion of ivermectin and subsequently tested human immunodeficiency virus (HIV) positive.

## Case presentation

A 38 year old Cameroonian married male, presented to our health service with facial rash, oral mucosa sores and discharging eyes of 2 days duration.

He reported being apparently well till 3 days prior to presentation when he ingested ivermectin 12 mg per os single dose served during a nationwide anti-filarial campaign. A day later (2 days prior to presentation), he noticed blisters on his lips which later extended to involve his oral mucosa. This was associated with pains on chewing and soreness of his entire oral mucosa which impaired feeding. Rashes developed on his face simultaneously. The rashes were itchy, scaly and extending towards the hairline. A day prior to consultation, his eyes became reddish and discharged clear fluid. The eyes were itchy and painful. The patient did not have fever and there were no other associated symptoms. Inability to feed well and his disfigured face prompted consultation in our health facility.

On reviewing his past medical history, he denied taking other medications including paracetamol prior to the onset of the symptoms. He however admitted taking a similar dose of ivermectin 1 year earlier during a similar campaign with no reactions. His wife was HIV positive and on highly active antiretroviral therapy. He reported that he was last tested for HIV 6 months prior to presentation and the test was negative. He did not provide any record of the test results. He had no known chronic illness and denied having any known drug or food allergies.

**Fig. 1:**
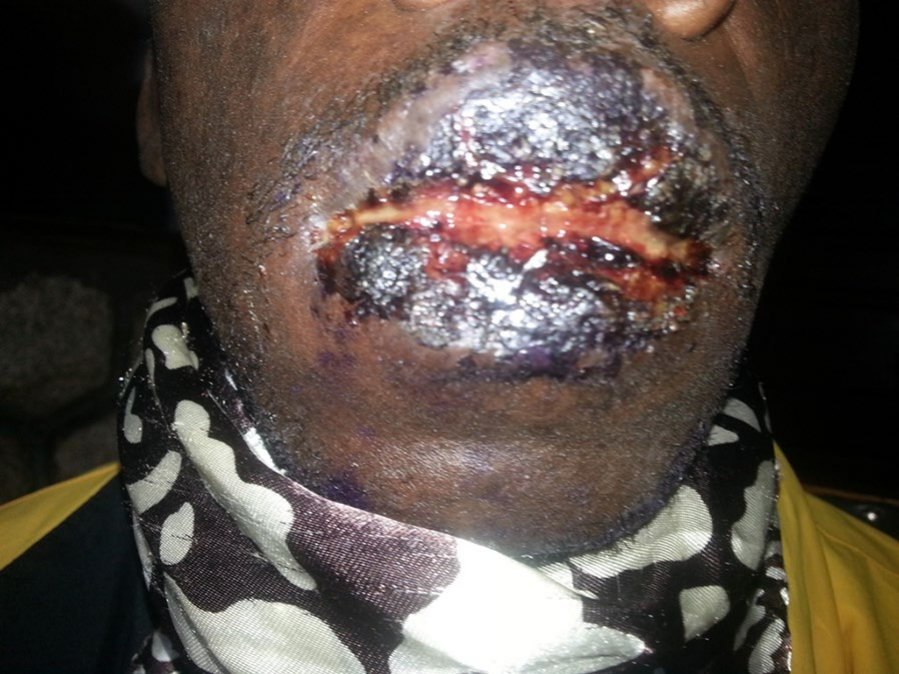
Photo of lesions on the lips

**Fig. 2:**
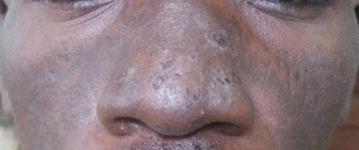
Photo of facial rash

On examination he was ill looking and in painful distress. There was a scarf around his face covering the oral lesions. The conjunctivae and sclerae were reddish and there was no cervical lymphadenopathy. His vital signs were within normal limits; blood pressure was 118/70 mmHg, heart rate; 84 beats/min, respiratory rate; 20 breaths/min, temperature; 37.2 °C, O_2_ saturation; 98% and weight 64 kg. There were desquamating hyper-pigmented rashes on his face with whitish plaques. The rashes were on the nasal bridge and extended to the malar area, sparing the nostrils. There were black eschars and erythematous erosions on the lips with sores and blisters in the oral mucosa (Figs. 1, 2). His eyes were erythematous, tearing and had sticky secretions that made his eyelids difficult to separate. There were no other skin lesions and the rest of his examination was normal.

Based on the clinical picture, a diagnosis of SJS was made with a differential of Lyell’s syndrome. Patient was counselled and tested for HIV. His test result was positive. A repeat 1st line test and second line test confirmed HIV seropositivity. Following HIV seropositivity, a CD4 count done to evaluate his immunological status was 568 cells/mm^3^. Other baseline tests revealed normal full blood count, liver enzymes and kidney function tests.

His management was mainly supportive: steroids (Dexamethasone 4 mg intramuscular route single dose, then prednisone 5 mg tablet: 4 tablets twice daily for 7 days then 2 tablets twice daily for 5 days and 1 tablet twice daily for 3 days), oral antihistamines (chlorpheniramine 4 mg tablets: 1 tablet twice daily for 10 days), antibiotic eye drops (ciprofloxacine eye drop: 1 drop on both eyes twice daily for 10 days), oral hygiene and nutritional support. He was reviewed a week later with much improved lesions and on week 3, most of the lesions had resolved.

## Discussion

Adverse drug reactions (ADRs) are on the rise, being responsible for about 6% of hospital admissions [10]. Cutaneous adverse drug reactions (CADRs) such as skin rashes, itches, urticaria, exfoliative dermatitis, Stevens–Johnson syndrome and Lyell’s syndrome are the most common manifestations of ADRs [11, 12]. SJS which is defined by an involvement of less than 10% of the epidermis, is a rare but potentially fatal form of CADR with a mortality of 5–15% [1, 11, 13]. Though SJS is thought to have varied causes, medications are most commonly implicated [2, 14]. Amongst medications, antimicrobials are most commonly associated with SJS [4].

Several antimicrobials have been implicated in SJS, with varying severities. Those with strong associations include sulphonamides (sulphadoxine, sulphadiazine, sulphasalazine and cotrimoxazole) and aminopenicillins (amoxicillin and ampicillin). While those which are less frequently associated SJS are: cephalosporins, floroquinolones, vancomicin, and antituberculose drugs (rifampicin and ethambutol) [11]. Cases of SJS have been reported with other antimicrobials not known to be typical causes of SJS such as fluconazole and streptomycin [6, 7]. A recent survey on the 25 year clinical use of ivermectin confirmed ivermectin to have a high margin of safety [15]. More so, a pubmed, google scholar, AJOL and HINARI search with keywords; ivermectin, SJS, side effects and case report showed that there was no preliminary report on ivermectin-induced SJS.

The mechanisms involved in the pathogenesis of SJS are still unclear, though reactive drug metabolites and immunological mechanisms have been suggested [16]. Reports suggest that metabolisms involved in the pathogenesis of CADRs involve interplay between activation and detoxification mechanisms. This imbalance may be as a result of inherited or acquired deficiency in enzymes required for detoxification. Also CADRs may result from increased levels of isoforms of cytochrome P450 responsible for obtaining drug metabolites from associated drugs. Increased level of substances serving as possible immunogens or cytotoxic agents may thus result from these metabolic discrepancies [17].

Underlying diseases, especially those that impair immunity such as infection with HIV, may also have an inducing role in the development of SJS [2, 18]. HIV seropositive patients have a higher incidence of SJS [14]. Several mechanisms have been proposed to explain the high incidence of CADRs in patients with HIV amongst which is glutathione deficiency, as glutathione conjugation of reactive metabolites and subsequent excretion is impaired [17]. Accumulated levels of these metabolites may therefore account for the higher occurrence of CADRs in HIV patients.

Ivermectin an extract of the fungus streptomyces avermitilis, is a semi-synthetic macrolide antibiotic of the class avermectin. It acts by attaching to glutamate-activated chloride channels, leading to increased influx of chloride [19]. Ivermectin is recommended for the treatment of onchocerciasis, cutaneous larva migrans and strongyloidosis [20]. It has also been shown to be efficacious in the management of scabies and myiasis [21, 22]. Onchocerciasis still remains a major problem in African countries [23, 24]. The Cameroon government in line with the world health organization’s African Programme for Onchocerciasis Control recommendations [25], intermittently provides ivermectin to its citizens as a mean of controlling and eliminating onchocerciasis. Ivermectin is metabolized by cytochrome P450 enzymes particularly the isozymes CYP3A4 and less so by the isozymes CYP2D6 and CYP2E1 [26]. We could therefore assume that cytochrome P450 metabolic idiosyncracies and/or deficiency of glutathione secondary to HIV infection are the main mechanisms responsible for this CADR.

To the best of our knowledge ivermectin has not been implicated as a cause of SJS in medical literature. That notwithstanding, clarithromycin another macrocyclic lactone (macrolide) though not of the avermectin family has been reported as a cause of CADRs [5]. Table 1 compares our patient to the patient with clarithromycin induced CADRs.

**Table 1:** Comparison of clinical profile and outcome in patients with macrolide induced CADRs

Characteristics	Ivermectin (our patient)	Clarithromycin [5]
Age	38 years	20 years
Gender	Male	Female
Type of reaction	SJS	Lyell’s syndrome
HIV status	Positive	Negative
Complication	None	Septic shock
Outcome	Lesions resolved and patient alive	Lesions persisted and patient died

Though controversial clinical profiles and outcomes, SJS is a possible side effect of macrolides, avermectins being inclusive.

The patient in this report had previously ingested ivermectin at same dose with no reaction. He however developed CADRs only following present intake. This may suggest that SJS here is idiosyncratic (dose independent) [27]. A year earlier during previous ingestion of ivermectin the patient was HIV negative (HIV negative test 6 months prior to consultation, though undocumented). He however developed SJS during subsequent ingestion of the drug following which he had seropositivity of HIV. There might thus be a link between HIV infection and ivermectin induced SJS.

## Conclusion

The case presented suggests that ivermectin is a possible cause of SJS which is a potentially life threatening complication of CADRs. Even though HIV increases risk of SJS, it remains unclear whether SJS in this patient was solely caused by ivermectin or by ivermectin influenced by concomitant HIV infection. The general population and in particular HIV infected individuals should be informed of this potentially fatal complication prior to and during such mass campaigns. Health workers should remain vigilant during such events.

## Abbreviations

SJS: Stevens–Johnson syndrome
CADRs: cutaneous adverse drug reactions
HIV: human immunodeficiency virus
